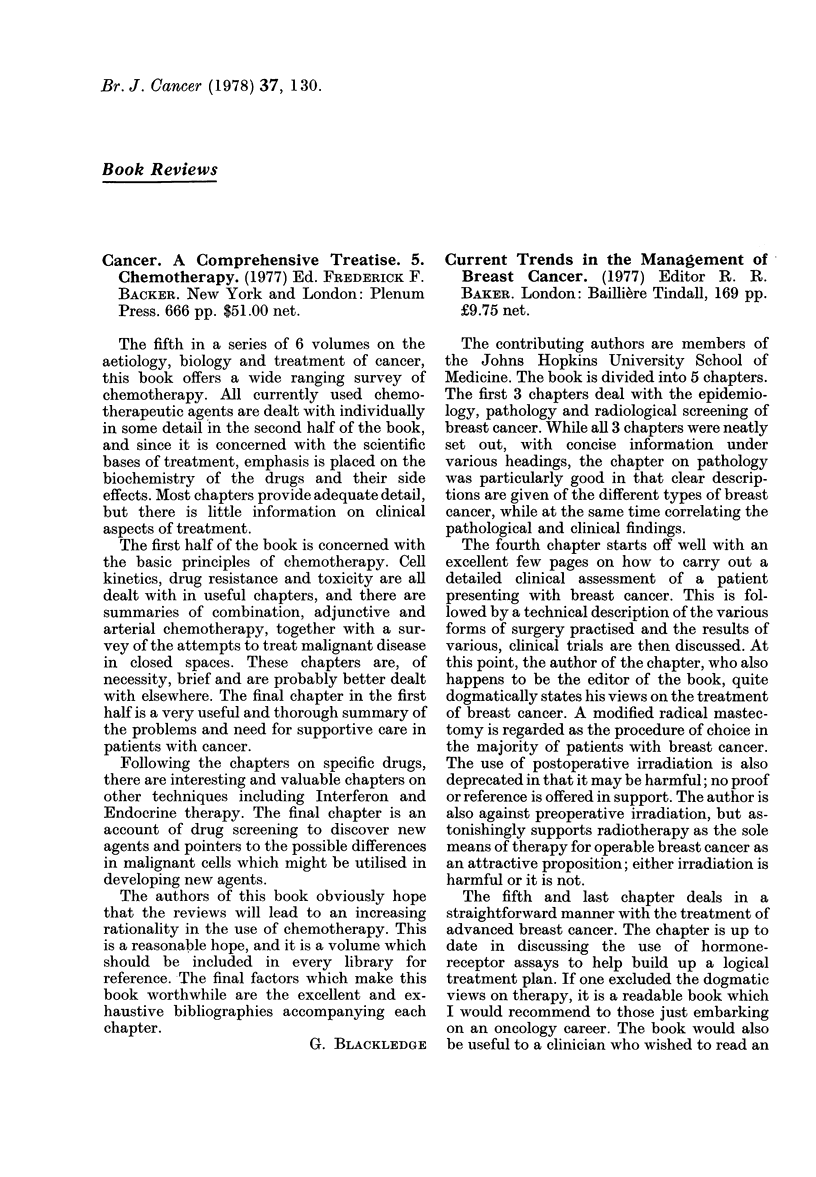# Cancer. A Comprehensive Treatise. 5. Chemotherapy

**Published:** 1978-01

**Authors:** G. Blackledge


*Br. J. Cancer* (1978) **37**, 130.

### *Book Reviews*

**Cancer. A Comprehensive Treatise. 5. Chemotherapy.** (1977) Ed. FREDERICK F. BACKER. New York and London: Plenum Press. 666 pp. $51.00 net.

The fifth in a series of 6 volumes on the aetiology, biology and treatment of cancer, this book offers a wide ranging survey of chemotherapy. All currently used chemotherapeutic agents are dealt with individually in some detail in the second half of the book, and since it is concerned with the scientific bases of treatment, emphasis is placed on the biochemistry of the drugs and their side effects. Most chapters provide adequate detail, but there is little information on clinical aspects of treatment.

The first half of the book is concerned with the basic principles of chemotherapy. Cell kinetics, drug resistance and toxicity are all dealt with in useful chapters, and there are summaries of combination, adjunctive and arterial chemotherapy, together with a survey of the attempts to treat malignant disease in closed spaces. These chapters are, of necessity, brief and are probably better dealt with elsewhere. The final chapter in the first half is a very useful and thorough summary of the problems and need for supportive care in patients with cancer.

Following the chapters on specific drugs, there are interesting and valuable chapters on other techniques including Interferon and Endocrine therapy. The final chapter is an account of drug screening to discover new agents and pointers to the possible differences in malignant cells which might be utilised in developing new agents.

The authors of this book obviously hope that the reviews will lead to an increasing rationality in the use of chemotherapy. This is a reasonable hope, and it is a volume which should be included in every library for reference. The final factors which make this book worthwhile are the excellent and exhaustive bibliographies accompanying each chapter.

G. BLACKLEDGE